# Editorial: Autobiographical memory, narrative skills, self processes, and individual differences: experimental, clinical, and forensic implications

**DOI:** 10.3389/fpsyg.2025.1594281

**Published:** 2025-04-09

**Authors:** Tiziana Maiorano, Luca Simione, Seungjin Lee, Monia Vagni

**Affiliations:** ^1^Department of Humanities, University of Urbino, Urbino, Italy; ^2^Dipartimento di Scienze Umane e Sociali Internazionali, Università degli Studi Internazionali di Roma (UNINT), Rome, Italy; ^3^Istituto di Scienze e Tecnologie della Cognizione, National Research Council (CNR), Rome, Italy; ^4^Department of Sang-Huh College, Konkuk University, Seoul, Republic of Korea; ^5^Department of Philosophy, Social Sciences, Humanities and Education, University of Perugia, Perugia, Umbria, Italy

**Keywords:** autobiographical memory, narrative skills, self processes, and individual differences: experimental, clinical, and forensic implications

Autobiographical memory is the ability to reconstruct and access personal memories (Conway, 2005; Rubin, 2005, 2019) and is a process involved in several important clinical and forensic application contexts such as eyewitness testimony and psychotherapy (Arterberry, 2022; Bauer, 2015). Knowing the mechanisms involved in the construction of autobiographical memories and identifying the factors of distortion and enhancement is of extreme importance for clinicians and forensic experts, who carry out their work both in the developmental field and for the evaluation of adults (Vagni et al., 2024). The main objective of this research topic was to analyze emotional, cognitive and situational factors that can distort the construction and recall of reliable and accurate autobiographical memories, such as stress, suggestions, and psychopathologies. Analyzing tools for assessing autobiographical memory in children and adults and testing the effectiveness of protocol for collecting memories were other important objectives. The Research Topic of articles published on this topic has explored several different contexts, broadening the reflection on it and opening up different ideas for reasoning and the construction of potential questions for future in-depth research. Some of the articles focused on in-depth analysis of aspects of autobiographical memory in the forensic field, presenting the relationship between autobiographical aspects and crime recidivism (Elias and Krackow), autobiographical narrative capacity and suggestibility in children (Vagni et al.; Lee and Shin) and analyzing evaluation protocols and presenting tools for measuring the implicit aspects of autobiographical memory linked to the guilt of a crime (Zangrossi et al.).

An article (Rossi-Arnaud et al.) instead presented the analysis of a method for recall of memories based on contact tracking used during the COVID pandemic, while another instead reflected about how emotional valence positive and negative of autobiographical memories, are central in the construction of identity (Pociunaite and Zimprich). Specifically Zangrossi et al. analyzed an important aspect of autobiographical memory in order to observe how methodologies for detecting implicit autobiographical memory can be useful in the forensic context within criminal trials. This research studied one of the behavioral memory detection methods, the autobiographical implicit association test (aIAT). The results of it are interesting because showed how participants who had pleaded guilty presented opposite patterns to innocent participants. Also they indicated that in the aIAT a greater fixation of category labels could indicate an increase in cognitive load and monitoring of response conflicts more present in people who plead guilty. Another article that analyzed the functioning of autobiographical narrative skills in the forensic field is the study by Vagni et al. that proposed a new tool to reliably evaluate autobiographical skills that can be useful for evaluation of children who are involved as witnesses or victims in criminal courts, context where it is necessary to understand autobiographical and narrative skills of children. This instrument is the Children Recalling Autobiographical Memory (CRAM) for measuring the main autobiographical narrative skills (where, what, when, who, and how) in relation to both retrospective and prospective memory, and it showed good internal reliability. Furthermore narrative autobiographical skills have been linked to suggestibility measured with the Gudjonsson Suggestibility Scale 2 (GSS2). Lee and Shin instead compared the effects of the narrative elaboration technique (NET) and openended rapport on accuracy and suggestibility of children's recall according to age. They found that open-ended rapport had a more positive impact on younger children's memory when compared to NET, while no differences emerged in older children's memory performance. These three articles focused on forensic evaluation tools achieving the objective of this topic about implementing research that has practical implications for evaluation of autobiographical memory. Elias and Krackow instead proposed a conceptual analysis of the literature on the topic of recidivism by comparing self-defined memories, which are specific types of autobiographical memory, in individuals not involved in justice and justice as in offenders. In particular, the authors focused on the model that explains recidivism by integrating self-defining memories with identity, the decision-making process and the behavioral processes underlying committing the same crime. The review is very interesting as it lays the theoretical foundation for future research approaches that can test the recidivism model. Knowing the mechanisms involved in the recidivism of a crime can be useful both for the purposes of social prevention and with a view to the rehabilitation of prisoners to work on modifying their concept of self which is defined by the construction of autobiographical memories and working toward a new identity. In conclusion, Pociunaite and Zimprich also analyzed the impact of autobiographical memory on the construction of identity, focusing however on the characteristics of positive and negative autobiographical memories (understood as emotional valence in terms of bipolar distinction and their conceptual independence) such as emotionality, vividness and the frequency with which a memory is retrieved and shared with others, as well as the ruminative and reflective focal points on the self.

Lastly, Rossi-Arnaud et al. analyzed the telephone interview tracing method used during the COVID-19 pandemic to trace contacts, in order to understand whether participants were able to provide useful information and how much their memories were influenced by events related to the early stages of the pandemic. This article highlights the importance of knowing the functioning of autobiographical memory by focusing on a method as tracing contact with a view to building future protocols that can be effective in possible scenarios of new pandemic emergencies. All articles, although very different in the topics addressed, fit together like pieces of a puzzle which together can add new reflections on the functioning of autobiographical memory and indicating practical application ideas. In particular Vagni et al. and Lee and Shin not only suggest the importance of avoiding leading questions in a forensic context but underline the importance of the use of accurate analysis of memories. Results of Elias and Krackow and Pociunaite and Zimprich indicate the importance to investigate the mechanism of identity. Finally Rossi-Arnaud et al. suggest the importance of using structured protocol for reconstruction of autobiographic memories. Lastly Zangrossi et al. suggest use of methodologies for detecting implicit autobiographical memory.
